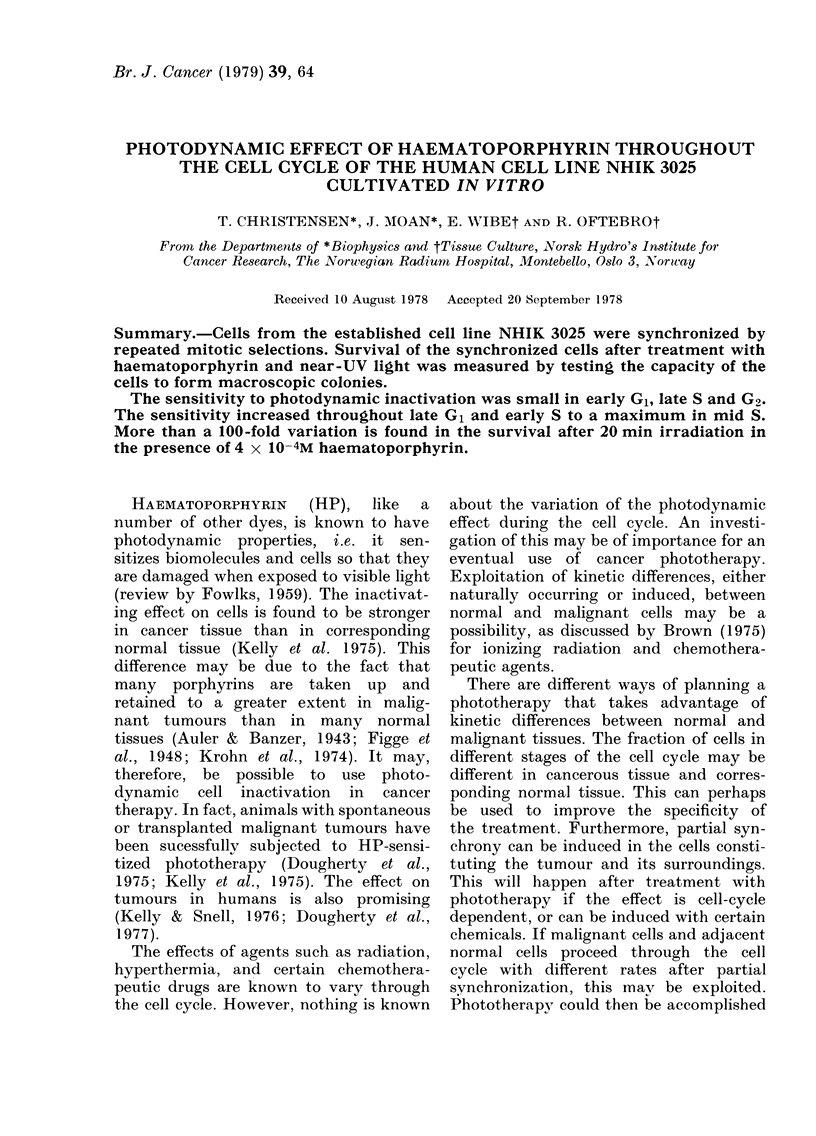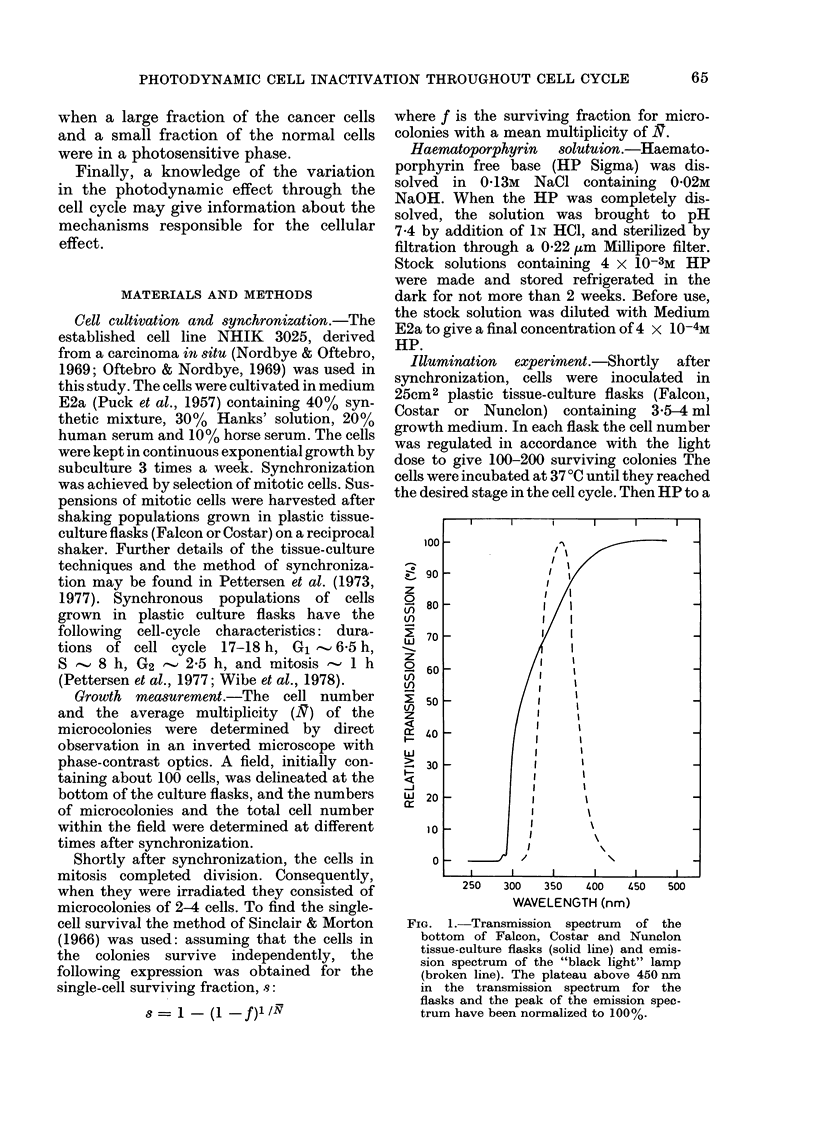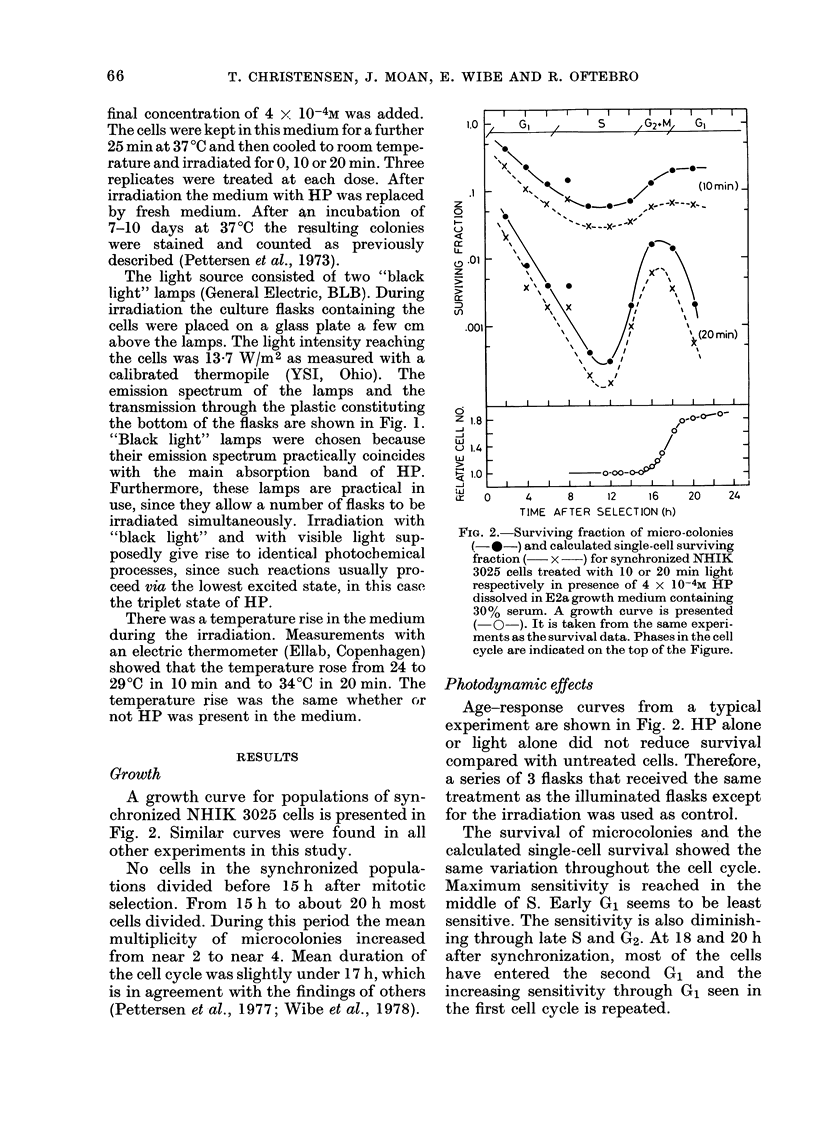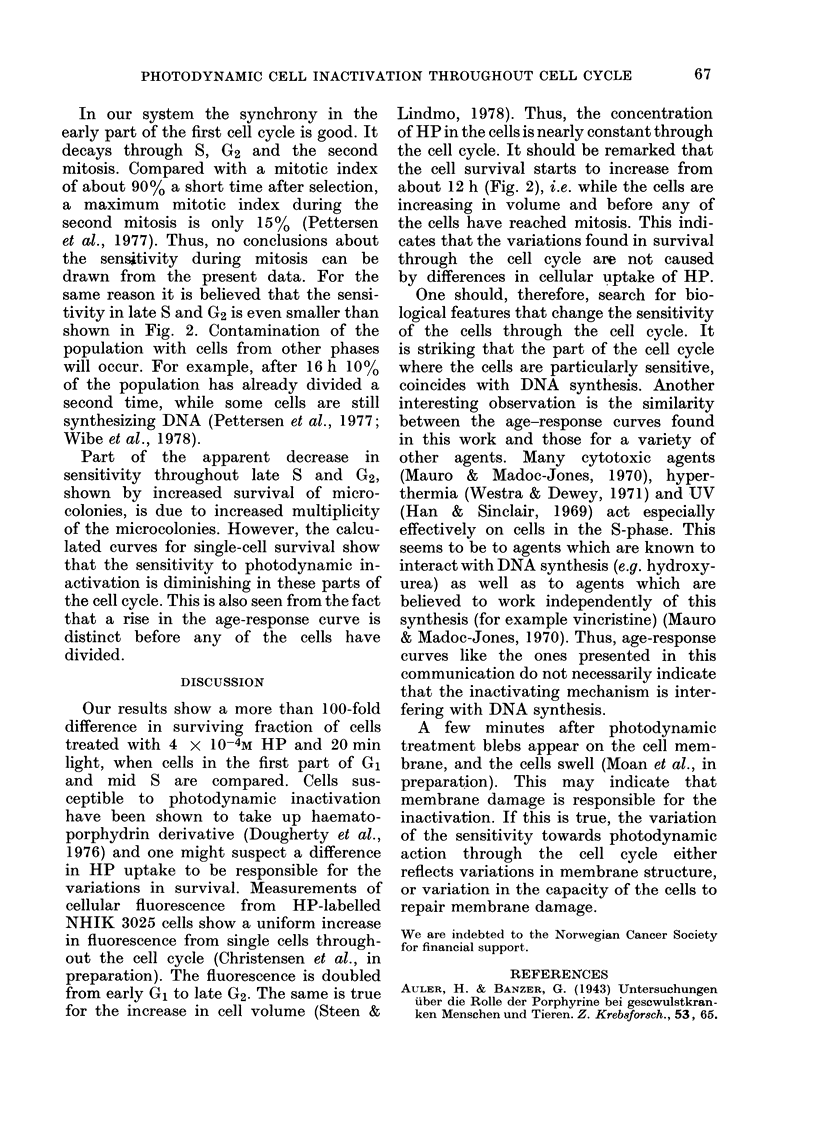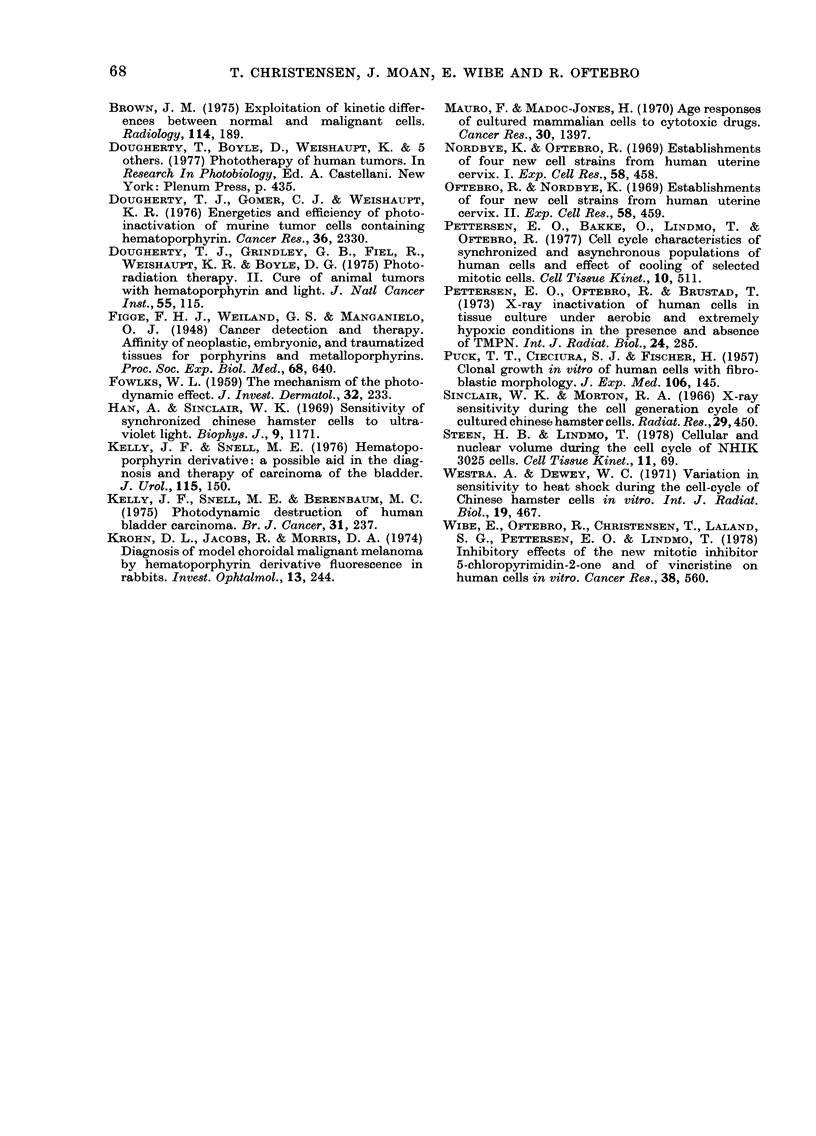# Photodynamic effect of haematoporphyrin throughout the cell cycle of the human cell line NHIK 3025 cultivated in vitro.

**DOI:** 10.1038/bjc.1979.8

**Published:** 1979-01

**Authors:** T. Christensen, J. Moan, E. Wibe, R. Oftebro

## Abstract

Cells from the established cell line NHIK 3025 were synchronized by repeated mitotic selections. Survival of the synchronized cells after treatment with haematoporphyrin and near-UV light was measured by testing the capacity of the cells to form macroscopic colonies. The sensitivity to photodynamic inactivation was small in early G1, late S and G2. The sensitivity increased throughout late G1 and early S to a maximum in mid S. More than a 100-fold variation is found in the survival after 20 min irradiation in the presence of 4 X 10(-4)M haematoporphyrin.


*Br. J. Cancer* (1979) **39**, 64

### PHOTODYNAMIC EFFECT OF HAEMATOPORPHYRIN THROUGHOUT THE CELL CYCLE OF THE HUMAN CELL LINE NHIK 3025 CULTIVATED *IN VITRO*

T. CHRISTENSEN*, J. MOAN*, E. WIBE† AND R. OFTEBRO†

*From the Departments of *Biophysics and †Tissue Culture, Norsk Hydro's Institute for Cancer Research, The Norwegian Radium Hospital, Montebello, Oslo 3, Norway*

Received 10 August 1978 Accepted 20 September 1978

**Summary.—Cells from the established cell line NHIK 3025 were synchronized by repeated mitotic selections. Survival of the synchronized cells after treatment with haematoporphyrin and near-UV light was measured by testing the capacity of the cells to form macroscopic colonies.**

**The sensitivity to photodynamic inactivation was small in early $G_1$, late S and $G_2$. The sensitivity increased throughout late $G_1$ and early S to a maximum in mid S. More than a 100-fold variation is found in the survival after 20 min irradiation in the presence of $4 \times 10^{-4}$M haematoporphyrin.**

HAEMATOPORPHYRIN (HP), like a number of other dyes, is known to have photodynamic properties, *i.e.* it sensitizes biomolecules and cells so that they are damaged when exposed to visible light (review by Fowlks, 1959). The inactivating effect on cells is found to be stronger in cancer tissue than in corresponding normal tissue (Kelly *et al.* 1975). This difference may be due to the fact that many porphyrins are taken up and retained to a greater extent in malignant tumours than in many normal tissues (Auler & Banzer, 1943; Figge *et al.*, 1948; Krohn *et al.*, 1974). It may, therefore, be possible to use photodynamic cell inactivation in cancer therapy. In fact, animals with spontaneous or transplanted malignant tumours have been sucessfully subjected to HP-sensitized phototherapy (Dougherty *et al.*, 1975; Kelly *et al.*, 1975). The effect on tumours in humans is also promising (Kelly & Snell, 1976; Dougherty *et al.*, 1977).

The effects of agents such as radiation, hyperthermia, and certain chemotherapeutic drugs are known to vary through the cell cycle. However, nothing is known about the variation of the photodynamic effect during the cell cycle. An investigation of this may be of importance for an eventual use of cancer phototherapy. Exploitation of kinetic differences, either naturally occurring or induced, between normal and malignant cells may be a possibility, as discussed by Brown (1975) for ionizing radiation and chemotherapeutic agents.

There are different ways of planning a phototherapy that takes advantage of kinetic differences between normal and malignant tissues. The fraction of cells in different stages of the cell cycle may be different in cancerous tissue and corresponding normal tissue. This can perhaps be used to improve the specificity of the treatment. Furthermore, partial synchrony can be induced in the cells constituting the tumour and its surroundings. This will happen after treatment with phototherapy if the effect is cell-cycle dependent, or can be induced with certain chemicals. If malignant cells and adjacent normal cells proceed through the cell cycle with different rates after partial synchronization, this may be exploited. Phototherapy could then be accomplished when a large fraction of the cancer cells and a small fraction of the normal cells were in a photosensitive phase.

Finally, a knowledge of the variation in the photodynamic effect through the cell cycle may give information about the mechanisms responsible for the cellular effect.

### MATERIALS AND METHODS

*Cell cultivation and synchronization.*—The established cell line NHIK 3025, derived from a carcinoma *in situ* (Nordbye & Oftebro, 1969; Oftebro & Nordbye, 1969) was used in this study. The cells were cultivated in medium E2a (Puck *et al.*, 1957) containing 40% synthetic mixture, 30% Hanks' solution, 20% human serum and 10% horse serum. The cells were kept in continuous exponential growth by subculture 3 times a week. Synchronization was achieved by selection of mitotic cells. Suspensions of mitotic cells were harvested after shaking populations grown in plastic tissue-culture flasks (Falcon or Costar) on a reciprocal shaker. Further details of the tissue-culture techniques and the method of synchronization may be found in Pettersen *et al.* (1973, 1977). Synchronous populations of cells grown in plastic culture flasks have the following cell-cycle characteristics: durations of cell cycle 17–18 h, $G_1 \sim 6{\cdot}5$ h, S $\sim$ 8 h, $G_2 \sim 2{\cdot}5$ h, and mitosis $\sim$ 1 h (Pettersen *et al.*, 1977; Wibe *et al.*, 1978).

*Growth measurement.*—The cell number and the average multiplicity ($\bar{N}$) of the microcolonies were determined by direct observation in an inverted microscope with phase-contrast optics. A field, initially containing about 100 cells, was delineated at the bottom of the culture flasks, and the numbers of microcolonies and the total cell number within the field were determined at different times after synchronization.

Shortly after synchronization, the cells in mitosis completed division. Consequently, when they were irradiated they consisted of microcolonies of 2–4 cells. To find the single-cell survival the method of Sinclair & Morton (1966) was used: assuming that the cells in the colonies survive independently, the following expression was obtained for the single-cell surviving fraction, *s*:

$$s = 1 - (1 - f)^{1/\bar{N}}$$

where $f$ is the surviving fraction for microcolonies with a mean multiplicity of $\bar{N}$.

*Haematoporphyrin solutuion.*—Haematoporphyrin free base (HP Sigma) was dissolved in 0·13M NaCl containing 0·02M NaOH. When the HP was completely dissolved, the solution was brought to pH 7·4 by addition of 1N HCl, and sterilized by filtration through a 0·22 $\mu$m Millipore filter. Stock solutions containing $4 \times 10^{-3}$M HP were made and stored refrigerated in the dark for not more than 2 weeks. Before use, the stock solution was diluted with Medium E2a to give a final concentration of $4 \times 10^{-4}$M HP.

*Illumination experiment.*—Shortly after synchronization, cells were inoculated in 25cm² plastic tissue-culture flasks (Falcon, Costar or Nunclon) containing 3·5–4 ml growth medium. In each flask the cell number was regulated in accordance with the light dose to give 100–200 surviving colonies The cells were incubated at 37 °C until they reached the desired stage in the cell cycle. Then HP to a

FIG. 1.—Transmission spectrum of the bottom of Falcon, Costar and Nunclon tissue-culture flasks (solid line) and emission spectrum of the "black light" lamp (broken line). The plateau above 450 nm in the transmission spectrum for the flasks and the peak of the emission spectrum have been normalized to 100%.

final concentration of $4 \times 10^{-4}$M was added. The cells were kept in this medium for a further 25 min at 37 °C and then cooled to room temperature and irradiated for 0, 10 or 20 min. Three replicates were treated at each dose. After irradiation the medium with HP was replaced by fresh medium. After an incubation of 7–10 days at 37 °C the resulting colonies were stained and counted as previously described (Pettersen *et al.*, 1973).

The light source consisted of two "black light" lamps (General Electric, BLB). During irradiation the culture flasks containing the cells were placed on a glass plate a few cm above the lamps. The light intensity reaching the cells was 13·7 W/m$^2$ as measured with a calibrated thermopile (YSI, Ohio). The emission spectrum of the lamps and the transmission through the plastic constituting the bottom of the flasks are shown in Fig. 1. "Black light" lamps were chosen because their emission spectrum practically coincides with the main absorption band of HP. Furthermore, these lamps are practical in use, since they allow a number of flasks to be irradiated simultaneously. Irradiation with "black light" and with visible light supposedly give rise to identical photochemical processes, since such reactions usually proceed *via* the lowest excited state, in this case the triplet state of HP.

There was a temperature rise in the medium during the irradiation. Measurements with an electric thermometer (Ellab, Copenhagen) showed that the temperature rose from 24 to 29 °C in 10 min and to 34 °C in 20 min. The temperature rise was the same whether or not HP was present in the medium.

### RESULTS

### *Growth*

A growth curve for populations of synchronized NHIK 3025 cells is presented in Fig. 2. Similar curves were found in all other experiments in this study.

No cells in the synchronized populations divided before 15 h after mitotic selection. From 15 h to about 20 h most cells divided. During this period the mean multiplicity of microcolonies increased from near 2 to near 4. Mean duration of the cell cycle was slightly under 17 h, which is in agreement with the findings of others (Pettersen *et al.*, 1977; Wibe *et al.*, 1978).

FIG. 2.—Surviving fraction of micro-colonies (—●—) and calculated single-cell surviving fraction (—— × ——) for synchronized NHIK 3025 cells treated with 10 or 20 min light respectively in presence of $4 \times 10^{-4}$M HP dissolved in E2a growth medium containing 30% serum. A growth curve is presented (—○—). It is taken from the same experiments as the survival data. Phases in the cell cycle are indicated on the top of the Figure.

### *Photodynamic effects*

Age–response curves from a typical experiment are shown in Fig. 2. HP alone or light alone did not reduce survival compared with untreated cells. Therefore, a series of 3 flasks that received the same treatment as the illuminated flasks except for the irradiation was used as control.

The survival of microcolonies and the calculated single-cell survival showed the same variation throughout the cell cycle. Maximum sensitivity is reached in the middle of S. Early $G_1$ seems to be least sensitive. The sensitivity is also diminishing through late S and $G_2$. At 18 and 20 h after synchronization, most of the cells have entered the second $G_1$ and the increasing sensitivity through $G_1$ seen in the first cell cycle is repeated.

In our system the synchrony in the early part of the first cell cycle is good. It decays through S, $G_2$ and the second mitosis. Compared with a mitotic index of about 90% a short time after selection, a maximum mitotic index during the second mitosis is only 15% (Pettersen *et al.*, 1977). Thus, no conclusions about the sensitivity during mitosis can be drawn from the present data. For the same reason it is believed that the sensitivity in late S and $G_2$ is even smaller than shown in Fig. 2. Contamination of the population with cells from other phases will occur. For example, after 16 h 10% of the population has already divided a second time, while some cells are still synthesizing DNA (Pettersen *et al.*, 1977; Wibe *et al.*, 1978).

Part of the apparent decrease in sensitivity throughout late S and $G_2$, shown by increased survival of microcolonies, is due to increased multiplicity of the microcolonies. However, the calculated curves for single-cell survival show that the sensitivity to photodynamic inactivation is diminishing in these parts of the cell cycle. This is also seen from the fact that a rise in the age-response curve is distinct before any of the cells have divided.

### DISCUSSION

Our results show a more than 100-fold difference in surviving fraction of cells treated with $4 \times 10^{-4}$M HP and 20 min light, when cells in the first part of $G_1$ and mid S are compared. Cells susceptible to photodynamic inactivation have been shown to take up haematoporphydrin derivative (Dougherty *et al.*, 1976) and one might suspect a difference in HP uptake to be responsible for the variations in survival. Measurements of cellular fluorescence from HP-labelled NHIK 3025 cells show a uniform increase in fluorescence from single cells throughout the cell cycle (Christensen *et al.*, in preparation). The fluorescence is doubled from early $G_1$ to late $G_2$. The same is true for the increase in cell volume (Steen & Lindmo, 1978). Thus, the concentration of HP in the cells is nearly constant through the cell cycle. It should be remarked that the cell survival starts to increase from about 12 h (Fig. 2), *i.e.* while the cells are increasing in volume and before any of the cells have reached mitosis. This indicates that the variations found in survival through the cell cycle are not caused by differences in cellular uptake of HP.

One should, therefore, search for biological features that change the sensitivity of the cells through the cell cycle. It is striking that the part of the cell cycle where the cells are particularly sensitive, coincides with DNA synthesis. Another interesting observation is the similarity between the age–response curves found in this work and those for a variety of other agents. Many cytotoxic agents (Mauro & Madoc-Jones, 1970), hyperthermia (Westra & Dewey, 1971) and UV (Han & Sinclair, 1969) act especially effectively on cells in the S-phase. This seems to be to agents which are known to interact with DNA synthesis (*e.g.* hydroxyurea) as well as to agents which are believed to work independently of this synthesis (for example vincristine) (Mauro & Madoc-Jones, 1970). Thus, age-response curves like the ones presented in this communication do not necessarily indicate that the inactivating mechanism is interfering with DNA synthesis.

A few minutes after photodynamic treatment blebs appear on the cell membrane, and the cells swell (Moan *et al.*, in preparation). This may indicate that membrane damage is responsible for the inactivation. If this is true, the variation of the sensitivity towards photodynamic action through the cell cycle either reflects variations in membrane structure, or variation in the capacity of the cells to repair membrane damage.

We are indebted to the Norwegian Cancer Society for financial support.

### REFERENCES

AULER, H. & BANZER, G. (1943) Untersuchungen über die Rolle der Porphyrine bei geschwulstkranken Menschen und Tieren. *Z. Krebsforsch.*, **53**, 65.

BROWN, J. M. (1975) Exploitation of kinetic differences between normal and malignant cells. *Radiology*, **114**, 189.

DOUGHERTY, T., BOYLE, D., WEISHAUPT, K. & 5 others. (1977) Phototherapy of human tumors. In *Research In Photobiology*, Ed. A. Castellani. New York: Plenum Press, p. 435.

DOUGHERTY, T. J., GOMER, C. J. & WEISHAUPT, K. R. (1976) Energetics and efficiency of photoinactivation of murine tumor cells containing hematoporphyrin. *Cancer Res.*, **36**, 2330.

DOUGHERTY, T. J., GRINDLEY, G. B., FIEL, R., WEISHAUPT, K. R. & BOYLE, D. G. (1975) Photoradiation therapy. II. Cure of animal tumors with hematoporphyrin and light. *J. Natl Cancer Inst.*, **55**, 115.

FIGGE, F. H. J., WEILAND, G. S. & MANGANIELO, O. J. (1948) Cancer detection and therapy. Affinity of neoplastic, embryonic, and traumatized tissues for porphyrins and metalloporphyrins. *Proc. Soc. Exp. Biol. Med.*, **68**, 640.

FOWLKS, W. L. (1959) The mechanism of the photodynamic effect. *J. Invest. Dermatol.*, **32**, 233.

HAN, A. & SINCLAIR, W. K. (1969) Sensitivity of synchronized chinese hamster cells to ultraviolet light. *Biophys. J.*, **9**, 1171.

KELLY, J. F. & SNELL, M. E. (1976) Hematoporphyrin derivative: a possible aid in the diagnosis and therapy of carcinoma of the bladder. *J. Urol.*, **115**, 150.

KELLY, J. F., SNELL, M. E. & BERENBAUM, M. C. (1975) Photodynamic destruction of human bladder carcinoma. *Br. J. Cancer*, **31**, 237.

KROHN, D. L., JACOBS, R. & MORRIS, D. A. (1974) Diagnosis of model choroidal malignant melanoma by hematoporphyrin derivative fluorescence in rabbits. *Invest. Ophtalmol.*, **13**, 244.

MAURO, F. & MADOC-JONES, H. (1970) Age responses of cultured mammalian cells to cytotoxic drugs. *Cancer Res.*, **30**, 1397.

NORDBYE, K. & OFTEBRO, R. (1969) Establishments of four new cell strains from human uterine cervix. I. *Exp. Cell Res.*, **58**, 458.

OFTEBRO, R. & NORDBYE, K. (1969) Establishments of four new cell strains from human uterine cervix. II. *Exp. Cell Res.*, **58**, 459.

PETTERSEN, E. O., BAKKE, O., LINDMO, T. & OFTEBRO, R. (1977) Cell cycle characteristics of synchronized and asynchronous populations of human cells and effect of cooling of selected mitotic cells. *Cell Tissue Kinet.*, **10**, 511.

PETTERSEN, E. O., OFTEBRO, R. & BRUSTAD, T. (1973) X-ray inactivation of human cells in tissue culture under aerobic and extremely hypoxic conditions in the presence and absence of TMPN. *Int. J. Radiat. Biol.*, **24**, 285.

PUCK, T. T., CIECIURA, S. J. & FISCHER, H. (1957) Clonal growth *in vitro* of human cells with fibroblastic morphology. *J. Exp. Med.* **106**, 145.

SINCLAIR, W. K. & MORTON, R. A. (1966) X-ray sensitivity during the cell generation cycle of cultured chinese hamster cells. *Radiat. Res.*, **29**, 450.

STEEN, H. B. & LINDMO, T. (1978) Cellular and nuclear volume during the cell cycle of NHIK 3025 cells. *Cell Tissue Kinet.*, **11**, 69.

WESTRA. A. & DEWEY, W. C. (1971) Variation in sensitivity to heat shock during the cell-cycle of Chinese hamster cells *in vitro*. *Int. J. Radiat. Biol.*, **19**, 467.

WIBE, E., OFTEBRO, R., CHRISTENSEN, T., LALAND, S. G., PETTERSEN, E. O. & LINDMO, T. (1978) Inhibitory effects of the new mitotic inhibitor 5-chloropyrimidin-2-one and of vincristine on human cells *in vitro*. *Cancer Res.*, **38**, 560.